# Mpox Infection in a Developed Country: A Case Report

**DOI:** 10.3390/tropicalmed8010015

**Published:** 2022-12-27

**Authors:** Tal Patalon, Galit Perez, Guy Melamed, Tamar Wolf, Sivan Gazit

**Affiliations:** 1 Kahn Sagol Maccabi (KSM) Research & Innovation Center, Maccabi Healthcare Services, Tel Aviv 68125, Israel; 2 Maccabitech Institute for Research and Innovation, Maccabi Healthcare Services, Tel Aviv 68125, Israel; 3 Department of Health Policy and Management, School of Public Health, Ben Gurion University of the Negev, Beer Sheva 84105, Israel; 4 Central Laboratory, Maccabi Healthcare Services, Rehovot 76703, Israel

**Keywords:** monkeypox, *Orthopoxvirus* genus, MPX, mpox

## Abstract

This is the first Israeli case report of mpox (monkeypox) disease, as it is manifested in the current outbreak. This manuscript depicts two detailed patient journeys of Israeli men in their 30s who were diagnosed in recent months, depicting their symptoms, presumed exposure, and outcomes. The two cases were atypical compared to the clinical presentation prior to the current outbreak but were similar to other recent reported cases; they differed in their prodromal presentation. Importantly, both patients described that significant anxiety around the diagnosis dominated their journey while sharing that a concern is rising in the GBMSM community, a concern that should be addressed by healthcare providers.

## 1. Introduction

Mpox (formerly denominated monkeypox) disease, caused by the zoonotic monkeypox virus of the Poxviridae family and the *Orthopoxvirus* genus, has evolved significantly since its diagnosis in humans in 1970. First, its incidence accelerated in recent years. During the 1970s, 47 cases were reported, whereas a decade later that number reached 356, without dramatic changes in the 1990s [1]. However, confirmed and suspected cases in the two decades between 2000 and 2019 increased to over 10,000 and 18,000, respectively, which was not considered to be related to improved reporting. The recent outbreak starting in May 2022 already includes 83,424 confirmed cases as of 22 December 2022 [2]. 

The second significant alteration was in the geographic distribution of mpox. After being detected in the Democratic Republic of Congo (DRC), mpox reached five other African countries during the 1970s, where a decade later it spread to four more countries in the continent. However, in 2003 the first infection was confirmed outside Africa, in a United States outbreak of 47 cases that were reported to have been exposed to infected animals but otherwise remained rather contained, with few non-African sporadic cases, such as one in Israel in 2018, four in the United Kingdom, and one in Singapore [1]. Conversely, the recent surge has spread to over 110 locations, with more than 90% of these areas reporting mpox for the first time. Unlike tens of thousands of cases in Europe and America, as of writing these lines Asia has only a few cases [2]. In Israel, 52 cases have been diagnosed thus far [3]. 

The clinical presentation of mpox has been altered as well, including a change in age at presentation, from young children to young adults. The current outbreak is seen mainly in young men who identify as gay or bisexual and other men who have sex with men (GBMSM). The mode of transmission has transformed as well, from animal-to-human transmission to human-to-human transmission [4].

## 2. Materials and Methods

The objective of this study is to describe patient journeys of mpox in Israel, as it is manifested in the current outbreak, through two detailed case reports while sharing detailed information as a lesson from the early days of the SARS-CoV-2 pandemic.

## 3. Case Reports

Both patients were Israeli men in their 30s who have sex with men (MSM) and were diagnosed through real-time PCR that assessed the presence of mpox in swabs taken from the oropharynx and the lesion. Neither had received the smallpox vaccine or were administered an antiviral treatment for mpox, and both reported self-quarantine after diagnosis, as was mandated at that time by the Israeli health regulators.

Patient 1 had a normal body mass index (BMI) and suffered from irritable bowel syndrome and hemorrhoids (Table 1). He also had a history of condyloma acuminatum a year prior to the current infection, followed by inoculation with the human papillomavirus (HPV) vaccine, the last dose of which was administered a month prior to infection. Additionally, he was diagnosed with chlamydia and gonorrhea in the past two years and was once infected and twice vaccinated against SARS-CoV-2 roughly a year before diagnosis. Patient 1 used a pre-exposure prophylaxis against HIV (PrEP).

In our investigation, a possible chain of transmission was identified. Patient 1 reported having unprotected sexual intercourse with his partner up to a day prior to symptom onset, while the partner (who was confirmed to be positive for mpox several days prior to patient 1) had previously engaged in unprotected intercourse with a traveler from Europe. No further transmissions were identified, and following diagnosis, patient 1 practiced self-quarantine and had no other close contacts.

The first reported symptom was a painless sensation of skin textural change in the perianal region, where no visible lesions were detected by a primary care physician. A day later (day 2) a clinical picture of viral infection ensued, with a low-grade fever of 37.5 degrees Celsius and fatigue. Given the exposure history and the patient’s own clinical suspicion of having been infected, the next visit with the primary care physician resulted in a referral to the emergency room (ER), following current Israeli guidelines for cases of suspected mpox. 

In the ER, the patient’s vital signs, apart from fever, were normal, and no additional areas of skin or mucosa were involved. Swabs from the anal lesions and nasopharynx were positive for mpox in a polymerase chain reaction (PCR) test. A complete blood count (CBC) and a biochemistry panel (including creatinine, electrolyte, and liver function tests) demonstrated slight neutrophilia of 6 × 10^3^/µL (normal range of 1.4–6 × 10^3^/µL) in the presence of normal leukocytes. C-reactive protein (CRP) was elevated to 93.43 mg/L (normal range of 0.03–5 mg/L) and alanine transaminase (ALT) reached 64 U/L (normal range of 8–39 U/L). Patient 1 was discharged with a topical antibiotic prescription and a recommendation to perform complete sexually transmitted disease panel testing in the community setting.

On day three, the measured fever rose to 38.0 degrees Celsius with chills, alongside muscle aches, a new severe headache, and bilateral tender inguinal lymphadenopathy. Additionally, the first lesions appeared, comprised of raised, painless, serous-secreting papules in the anal and perianal regions. On the fourth day of symptoms, the fever reached 38.6 Celsius, the lymphadenopathy was more severe, and a peak of inguinal pain was reported. The number of lesions amounted to 10, and a single papular lesion appeared in the oral commissure.

**Figure 1:**
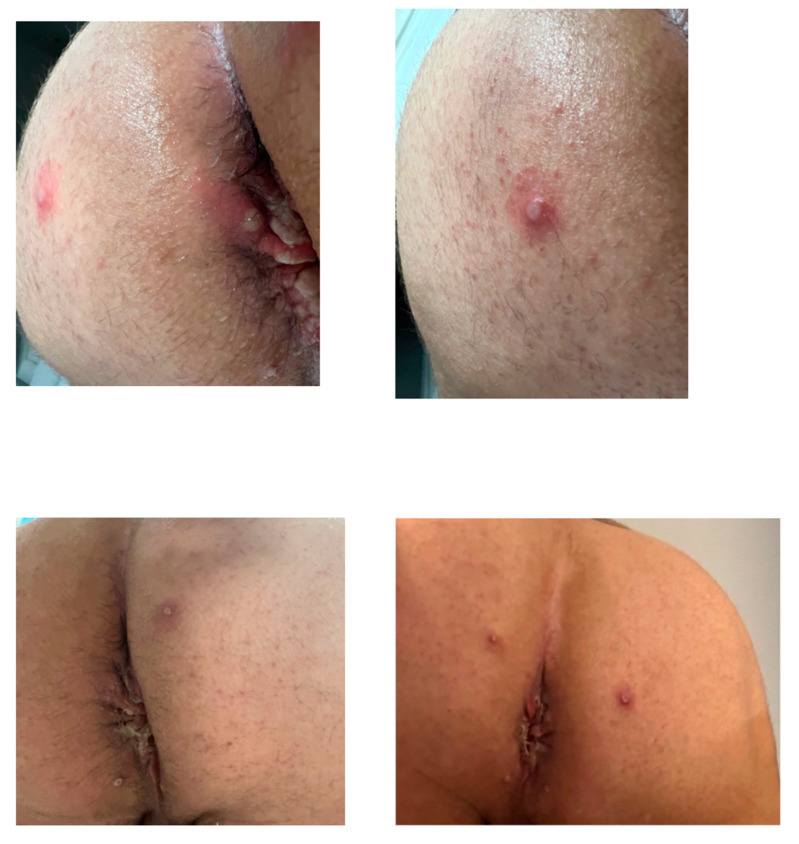
Lesions of perianal region, patient 1. Top row represents day 5 from symptom onset; bottom row was captured on day 8.

Day 5 of symptoms was characterized by some anal, perianal, and buttocks lesions, exhibiting central umbilication (Figure 1). Some of them progressed to pruritic pustules. Additionally, raised red patches spread over the neck, trunk, and upper extremities. The patient was treated with pain control medications, antihistamines, and topical creams. A day later, painless papules appeared on the trunk and upper extremities. 

On the 7th day, the fever broke, and the red patches subsided, but the anal lesions morphed into a vesicular appearance, causing pruritis, severe anal pain, and dyschezia, forcing the patient to seek emergency medical care that day. His second visit to the ER had similar laboratory results. The patient was discharged with a combination of Oxycodone and Paracetamol. On day 8, the lymphadenopathy and pain subsided, and some of the anal and perianal lesions began to crust, though pruritis was still prominent. On days 9 and 10, constitutional symptoms were insignificant, except fatigue, but the anal pain worsened alongside the growth of lesions there. A day later, the patient was prescribed Tramadol. On days 11 to 14, vesicles disappeared asynchronously, with hypopigmentation in previously affected areas, alongside a decrease in pruritis. On the 15th day, there were no visible lesions, apart from hypopigmentation, but a rough/protruding sensation was still present. From day 19 to day 60, there were no visible mucocutaneus changes, though the patient reported discomfort during defecation and anal intercourse. 

Importantly, throughout the first two weeks, patient 1 also reported a high anxiety level (maximal on a scale of 0 to 5), referring to the rapid changes in symptomatology and the unknown expected duration of the disease. Notably, the patient also mentioned that a high level of anxiety regarding mpox has characterized the gay community since the outbreak, including a feeling of needing to hide the condition in the community, mentioning that it feels similar to past attitudes towards HIV.

The second case refers to an HIV-positive male, treated with a combination of abacavir, dolutegravir, and lamivudine, with a CD4^+^ T-cell count of over 500 cells/mm^3^ (normal range of 436–1394). Patient 2 also used Apixaban, following a venous thromboembolism (VTE) event (Table 1). Similar to patient 1, patient 2 had a normal BMI, had a history of condyloma acuminatum, and was administered the HPV vaccine, with the last dose administered several weeks prior to infection.

The symptoms in patient 2 began two weeks after reported unprotected sexual intercourse with a traveler from Europe, and like patient 1, it was the patient himself who raised the first suspicion of mpox.

Day one of symptoms included malaise, dysuria and increased urinary frequency, penile pruritis (without visible lesions), and unilateral inguinal lymphadenopathy. Over the next two days, a single lesion with central umbilication was identified on the glans penis. On the fourth day, 10 asynchronous lesions comprised of pustules were identified over the penis as well as papules that spread to all bodily regions. On day 5, apart from lesions, cervical lymphadenopathy, severe headache, and significant fatigue were reported, and the patient was examined in the ER. There, vital signs, CBC, and a biochemistry panel (including creatinine, electrolytes, and liver function tests) as well as urinalysis were all within the normal ranges. 

Over the next four days, the number of lesions increased to about 20 lesions, which increased in their distribution, though sparing the face. The penile lesion secreted pus. On day 10, the lesions began to crust, and no other symptom was present. Some lesions completely disappeared by day 13, though the penile lesion was visible. The anxiety levels relating to the diagnosis remained significant, even as symptoms were improving, as the patient felt the disease was unknown and its course was unclear.

## 4. Discussion

This is the first Israeli case report of mpox disease in the current outbreak, and among the first in developed countries. We describe two patients, both men in their 30s who have sex with men (MSM), similar to the majority of reported cases around the world [5], reiterating the current debate of whether this new outbreak of mpox should be considered a sexually transmitted disease [6,7].

The two presented cases were atypical compared to the clinical pictures prior to the current outbreak, similar to other recent reported cases [8], as they included asynchronous lesions (lesions at different phases of development) over the anal and genital areas as well as over the trunk and extremities. This atypical classification matches the sporadic case reports that first came out of Italy [9] and Australia [10]. Additionally, the presence of lymphadenopathy in both patients is a classic feature of mpox, differentiating it from other pox diseases [11]. Nonetheless, the timing of the lymphadenopathy was different between the two cases, where in patient 1 it was a part of a ‘typical’ prodromal stage, while in patient 2 it was not present before the lesions. This lack of a defined prodromal stage has also been observed in other case reports [12] and represents the changing course of the disease [4]. The pervasiveness of the mucocutaneus manifestations of both patients matched the accumulated knowledge of the current outbreak, including the dominance of perianal and penile manifestations. The severe anal pain experienced by patient 1 is less prevalent [13], but when it is reported it often requires emergent medical attention, as was the case here. Neither patient received antiviral treatment or required hospitalization, and the disease was self-limiting [13]. Lastly, both patients described that significant anxiety around the diagnosis dominated their journey, while sharing that a concern is rising in the GBMSM community, a concern that should be specifically addressed by healthcare providers, a call for action that has been voiced in other parts of the world [14,15].

Given the rapid increase in positive cases, the threshold of suspicion should be lowered, alongside increasing the availability of testing and education about human mpox disease. It is important to inform suspected or diagnosed patients about transmission modes and the need for self-quarantine. Researchers should investigate the infectious period in order to determine the measures needed to minimize transmission. At the same time, we should address the issue of the emotional stress and anxiety that is emerging in the gay community [14]. Additionally, the medical community and healthcare policy makers should implement lessons learned from the SARS-CoV-2 pandemic, especially those relating to epidemiological investigations and contact tracing, maintaining a sufficient supply of tests, and providing rapid and open access to information around the world. Importantly, educational programs about emerging diseases, as they become more common, should be incorporated faster for medical professionals and trainees [16].

## 5. Conclusions

The threshold of suspicion should be lowered, alongside increasing the availability of testing and education about human mpox disease for both the public and the medical community itself. Importantly, the medical community should implement lessons learned from the SARS-CoV-2 pandemic, especially those relating to epidemiological investigations and contact tracing, rapid and open access to information around the world, and attention to vulnerable populations. In our case report, both patients described that significant anxiety around the diagnosis dominated their journey, while sharing that a concern is rising in the GBMSM community; a concern that should be specifically addressed by healthcare providers.

## Figures and Tables

**Table 1:** Summary of patient journeys.

	Patient 1	Patient 2
Demographics		
Sex	Male	Male
Age range	30–40	30–40
General medical history		
BMI range ^1^	18.5 to 24.9	18.5 to 24.9
IBS ^2^	Yes	No
Hemorrhoids	Yes	No
VTE ^3^	No	Yes
History of STIs ^4^		
HIV (CD4+ T-cell count, cells/mm^3^)	No	Yes (>500)
Condyloma Acuminatum	Yes	Yes
Chlamydia	Yes	No
Gonorrhea	Yes	No
Possible exposure vector	Unprotected sexual intercourse with an infected male partner a day prior to diagnosis	Unprotected sexual intercourse with a male traveler from Europe two weeks prior to diagnosis
Patient journey		
Minimal time from possible exposure to first symptom (days)	1	14
Duration of acute symptoms (days)	19	13
Types of reported symptoms		
Mucocutaneus lesions	Yes	Yes
Asynchronous lesions	Yes	Yes
Anatomical region of first lesion	anal and perianal regions	penis
Pain of lesions	Yes	No
Pruritis	Yes	Yes
Fever (maximum in Celsius)	Yes (38.6 °C)	No
Chills	Yes	No
Malaise	No	Yes
Fatigue	Yes	Yes
Headache	Yes	Yes
Myalgia	Yes	No
Dysuria	No	Yes
Lymphadenopathy (regions)	Yes (inguinal)	Yes (inguinal and cervical)
Lymphadenopathy before lesions	No	Yes
Anxiety	Yes	Yes

^1^ A body mass index (BMI) of 18.5 to 24.9 is considered to be within the normal range. ^2^ Irritable bowel disease. ^3^ Venous thromboembolism (VTE). ^4^ Sexually transmitted infections (STIs).

## Data Availability

According to the Israel Ministry of Health regulations, individual-level data cannot be shared openly. Specific requests for remote access to de-identified community-level data should be referred to KSM, Maccabi Healthcare Services Research and Innovation Center.
